# Brain imaging-wide exploration of site-specific pain: genetic correlation and Mendelian randomization study

**DOI:** 10.1017/S0033291726105091

**Published:** 2026-07-28

**Authors:** Weian Mao, Peipei Ye, Shuwei Wang, Mengchun Chen, Haiying Lv, Shuai Yuan, Fuhua Yan, Lili Yang

**Affiliations:** 1 Department of Obstetrics and Gynecology, The Second Affiliated Hospital of Wenzhou Medical University, China; 2 Department of Radiology, The Second Affiliated Hospital of Wenzhou Medical University, China; 3 Department of Anesthesia, Zhongshan Hospital, Fudan University, China; 4 Department of Pharmacy, The Second Affiliated Hospital of Wenzhou Medical University, China; 5 Department of Radiology, Ruijin Hospital, Shanghai Jiaotong University School of Medicine, China; 6 Unit of Cardiovascular and Nutritional Epidemiology, Institute of Environmental Medicine, Karolinska Institutet, China

**Keywords:** brain structure and function, Mendelian randomization, neuroimaging, pain

## Abstract

**Background:**

Observational studies have suggested that brain imaging-derived phenotypes (IDPs) may serve as specific markers of pain-related phenotypes and severity. However, the shared genetic architecture between pain and brain IDPs and their potential causal relationships remains unclear.

**Methods:**

We applied linkage disequilibrium score regression and Mendelian randomization (MR) analyses to uncover genetic correlations and potential causal links of brain structural (33,224 UK Biobank participants) and functional (47,276 UK Biobank participants) changes with site-specific pain phenotypes (approximately 500,000 Finngen participants). The scoping literature review was conducted to compare current findings with previous observational studies.

**Results:**

In this study, we identified 559 significant genetic correlations between 587 structural IDPs and 13 pain-related phenotypes. Using MR analyses, we found that genetic liability to headache, migraine, joint pain, and sciatica was causally associated with alterations in 15 structural IDPs. Additionally, changes in the surface area of three brain regions were linked to a lower risk of sciatica, low back pain, and overall pain. Among the six pain-related phenotypes associated with structural IDPs, further analyses demonstrated putative causal relationships between functional IDPs and these conditions. Notably, headache exhibited both significant structural and functional changes across three key brain regions: the superior frontal gyrus, lingual gyrus, and paracentral lobule.

**Conclusions:**

These findings provide novel insights into the genetic correlations and genetically inferred associations between pain and neurobiological abnormalities from neuroimaging perspectives, with structural alterations as the primary findings and functional changes as complementary exploratory evidence, advancing the understanding of pain-related mechanisms.

## Introduction

Pain represents a complex sensory and emotional phenomenon related to actual or potential tissue injury (Raja et al., 2020). It is one of the most common neurological conditions and a significant source of disability and suffering globally, imposing substantial personal and social burdens (Disease, Injury, & Prevalence, 2018). Pain patterns and severity can vary widely among individuals. Despite similar levels of tissue damage or neuropathy, different individuals may perceive pain differently, complicating pain management and treatment (Meng et al., 2023; Ploner, Bingel, & Wiech, 2015). Therefore, universally accepted objective indicators and further advancements in its fundamental mechanisms are critically needed.

The rapid development of neuroimaging techniques, exemplified by magnetic resonance imaging (MRI), has demonstrated the potential for objective measurements of brain anatomical structures and activity patterns that underlie perceptual experiences (Davis et al., 2017, 2020). Compelling structural MRI (sMRI) studies indicate that gray matter (GM) and white matter (WM) morphology are altered across various pain conditions, including migraine, low back pain, joint pain, and other chronic disorders. These alterations occur in multiple brain regions involved in sensory processing, affective modulation, and cognitive control, such as the somatosensory cortex, prefrontal cortex, cingulate cortex, insula, amygdala, hippocampus, and thalamus (Bhatt et al., 2024; Seifert et al., 2016; Selvarajah et al., 2023). Additionally, functional MRI (fMRI) analyses, a technique to capture changes in functional activity and connectivity within brain regions, have indicated that intrinsic connectivity within and between brain networks involved in pain processing and regulation is disrupted (Galambos et al., 2019; Mayr et al., 2022). As such, brain imaging-derived phenotypes (IDPs) provide a valuable framework for elucidating whether alterations in brain structure and function arise as a result of pain or contribute to its development (Messina, Rocca, Goadsby, & Filippi, 2023; Wang et al., 2023). This understanding could advance knowledge of the underlying neurobiological mechanisms and facilitate the application of neuroimaging in early prediction, diagnostic categorization, and therapeutic intervention strategies for pain. However, observational studies often face challenges with unmeasured and residual confounding factors that impede the establishment of causal relationships. First, most neuroimaging studies in pain research rely on relatively small, clinically ascertained samples, limiting generalizability and precluding causal inference. Second, conclusions across studies are often heterogeneous, with region-specific alterations varying by pain subtype, duration, and comorbidity profile (Kollenburg et al., 2026). Moreover, structural and functional imaging markers are frequently examined in isolation, and few studies have systematically integrated multimodal imaging phenotypes within a unified genetic framework. These uncertainties underscore the need for genetically informed approaches capable of disentangling shared genetic liability from directional causal effects across multimodal brain imaging traits and pain phenotypes.

To address these challenges, Linkage Disequilibrium Score (LDSC) regression and Mendelian randomization (MR) leverage genome-wide association study (GWAS) summary data to investigate associations between phenotypes. LDSC regression provides an estimate of shared genetic architecture between two phenotypes (B. K. Bulik-Sullivan et al., 2015). MR analyses strengthen causal inference regarding exposure-outcome associations by minimizing confounding and reverse causality (Burgess, Butterworth, & Thompson, 2013). Leveraging data from large-scale IDPs GWAS, researchers have uncovered substantial evidence of shared genetic architecture and potential causal relationships between brain imaging features and neuropsychiatric, neurodegenerative, and cardiovascular diseases (Guo et al., 2022; May-Wilson et al., 2022; Seyedsalehi et al., 2023; Yu et al., 2023). However, such work is still absent for IDPs and pain-related phenotypes.

In this study, we aimed to comprehensively investigate the genetic correlations and potential causalities between structural and functional IDPs and pain-related phenotypes. We first examined shared genetic basis using LDSC to identify significant correlations and then applied MR bidirectionally to assess whether changes in the brain’s structure and functional connectivity were causally associated with susceptibility to specific pain.

## Methods

### Data source for pain-related phenotypes

We collected genetic summary data for 13 pain-related phenotypes from the FinnGen study, which includes overall pain in major body parts (limbs, back, neck, head, and abdomen) and pain at specific sites (joint pain, limb pain, low back pain, female reproductive organ– and menstrual cycle–related pain, abdominal and pelvic pain, throat and chest pain, sciatica, headache, migraine, other headache syndromes including cluster headaches, ocular pain, and temporomandibular disorders [TMDs] muscular pain linked with fibromyalgia). These pain-related phenotypes were defined based on the International Classification of Diseases (ICD) codes (Supplementary Table S1) with data from the Finnish national registers. The number of cases for these 13 pain-related phenotypes ranged from 2,341 for ocular pain to 189,683 for overall pain (Kurki et al., 2023).

### Data source for brain structural IDPs

We used GWAS summary statistics of structural IDPs generated from a cohort of 33,224 European-descent participants in the UK Biobank (Smith et al., 2021). These IDPs included two measurements: sMRI and diffusion MRI (dMRI). sMRI captures variations in brain structure processed with FreeSurfer, such as cortical area, thickness, and volume, while dMRI measures structural connectivity, which reflects the physical connections of neural fibers between different regions of the brain using the diffusion tensor imaging (DTI) fitting tool DTIFIT and neurite orientation dispersion and density imaging (NODDI) tool AMICO (Accelerated Microstructure Imaging via Convex Optimization) (Smith et al., 2021). dMRI reported six types of measurements within multiple tract regions, comprising fractional anisotropy (FA), mean diffusivity (MD), diffusion tensor mode (MO), intracellular volume fraction (ICVF), isotropic or free water volume fraction (ISOVF), and orientation dispersion index (OD). For our analysis, we selected 587 brain structural IDPs with reliable segmentation and meaningful measurement from the 3,913 original releases, based on previous studies (Guo et al., 2022; Yu et al., 2023). Additionally, these IDPs were organized into 13 regional categories: 8 for brain anatomy and 5 for structural connectivity. GWAS summary statistics for structural IDPs were accessed through the Oxford Brain Imaging Genetics (BIG40) web browser (https://open.win.ox.ac.uk/ukbiobank/big40/). Additional summary information for these IDPs is presented in Supplementary Table S2, and a more detailed description of the GWAS can be found in the original GWAS paper (Smith et al., 2021).

### Data source for brain functional IDPs

For the pain-related phenotypes found to be associated with structural IDPs in either direction, we further investigated their relationships with functional network connections in various brain regions to better understand the potential neurological pathways linking pain and brain structure. To achieve this, we retrieved the available genetic summary statistics for 191 resting-state functional MRI (rsfMRI) measures generated by Zhao et al. (2022). The rsfMRI traits represent intrinsic functional connectivity measures derived from blood-oxygen-level-dependent (BOLD) signal correlations between predefined brain regions and major functional networks, including default mode, central executive, salience, somatomotor, attention, limbic, and visual networks from 47,276 UK Biobank participants (Supplementary Table S3). We then conducted bidirectional MR analyses for each pair of rsfMRI measures and pain-related phenotypes.

### Literature review of pain and IDPs

We conducted a scoping literature review for the interpretation of our findings. The research was performed using PubMed as sources, up to March 15, 2026. We searched for the terms of specific pain conditions and brain regions in combination with ‘magnetic resonance imaging’, ‘voxel-based morphometry’, ‘functional magnetic resonance imaging’, ‘voxel-based morphometry’, ‘gray matter’, ‘white matter’, ‘resting-state functional magnetic resonance imaging’, ‘regional homogeneity’, and ‘amplitude of low frequency fluctuation’. Review articles and the reference lists of relevant studies were also examined to identify potential omitted publications in the study. Only the literature pertaining to significant associations between IDPs and pain-related phenotypes observed in this study is included in the manuscript for presentation and discussion. All relevant articles were carefully reviewed and judged relevant to the topic. Finally, we extracted information on the characteristics of the patients and controls, sample size, and assessment metrics, which allowed us to summarize the direction of significant associations between IDPs and pain reported in clinical studies.

### Statistical analysis

First, we performed a genetic correlation analysis using the LDSC software to investigate the shared genetic basis that underlies each pair of structural IDP and pain-related phenotype (B. Bulik-Sullivan et al., 2015; B. K. Bulik-Sullivan et al., 2015). The pairs that showed statistically significant evidence of genetic correlation (*P* < 0.05) were then chosen for bidirectional MR analysis.

For the MR analysis, the same criteria were applied for IV selection when using either IDP or pain-related phenotype as the exposure. First, we excluded all palindromic single-nucleotide polymorphisms (SNPs) to prevent incorrect strand inference during data harmonization. Next, we performed clumping on the genetic summary data using the 1000 Genome European reference to select independent SNPs as IVs for each exposure. The clumping criteria included a significance threshold of *P* < 5 × 10^−8^, a linkage disequilibrium threshold of *r*^2^ < 0.01, and a clumping window size of 500 kb. For IVs absent from the outcome dataset, proxy SNPs were identified based on strong linkage disequilibrium (*r*^2^ > 0.80) and genome-wide significant associations with the exposure (*P* < 5 × 10^−8^). When multiple proxy SNPs were available, the one with the lowest *P*-value associated with the exposure was chosen for the analysis. The F statistic was estimated for each set of independent IVs to test their strength in representing the exposure, and a value typically less than 10 indicates weak IVs (Burgess &Thompson, 2011). We also conducted a sensitivity analysis by applying a stricter clumping window size of 10 Mb to minimize potential influence from related SNPs.

We used two-sample MR analyses to estimate the association between IDPs and pain-related phenotypes. When multiple IVs were available, causal inference was primarily performed using the inverse variance weighted (IVW) approach with a multiplicative random effects model (Burgess et al., 2013). In cases where only one IV was available, estimates were assessed using the Wald ratio method (Boehm & Zhou, 2022). The *P*-values from these two methods were used to identify significant MR results after a false discovery rate (FDR) correction for 1,107 tests conducted in both directions (FDR-adjusted *P* < 0.05). We also conducted sensitivity analyses using the weighted median method and the MR-Egger method (Boehm & Zhou, 2022). The weighted median method generates consistent causal estimates even if up to half of the IVs used are invalid. The MR-Egger method is robust even if all variants are invalid, and its intercept provides a test for unbalanced pleiotropy that violates the MR estimates (*P* for pleiotropy <0.05). We also tested the heterogeneity within the IVs using Cochran’s Q test (*P* for heterogeneity <0.05), which is a weaker indicator than the MR-Egger intercept for potential unbalanced pleiotropy (Boehm & Zhou, 2022). Mendelian randomization pleiotropy residual sum and outlier (MR-PRESSO) test was performed to identify potential outlying IVs and obtain estimates after removing the outliers (Verbanck, Chen, Neale, & Do, 2018). The same bidirectional MR analysis was applied to functional IDPs and pain-related phenotypes, and significant estimates with FDR-adjusted *P* < 0.05 were retained from the 2,292 tests conducted in both directions. To evaluate the robustness and generalizability of the identified associations, we performed a cross-population validation analysis using summary statistics from Asian populations (Fu et al., 2024; Karczewski et al., 2025). Specifically, MR analyses were conducted using headache as the exposure and the corresponding structural IDPs as outcomes, applying the same analytical framework as in the European dataset.

Since behavioral, sociocultural, and psychiatric factors can potentially complicate the association between brain structural alterations and pain phenotypes, we further examined each independent IV and its proxies (with *R*^2^ ≥ 0.8) for the IDP–pain pairs that showed significant associations in the PhenoScanner GWAS database with *P* for association <1 × 10^−5^ (Kamat et al., 2019; Staley et al., 2016). We assessed potential associations with smoking, drinking, socioeconomic status, educational attainment, and various psychiatric traits, including anorexia nervosa, anxiety, attention deficit hyperactivity disorder, autism spectrum disorder, bipolar disorder, major depressive disorder, neuroticism, and schizophrenia. We conducted a sensitivity analysis excluding these pleiotropic IVs to avoid the observed associations being driven by potential pleiotropic effects. In addition, multivariable MR analyses were conducted to estimate the direct effect of structural IDPs on pain, accounting for potential confounders or mediators (Clarke et al., 2017; Hemani et al., 2018; Liu et al., 2019; Loh et al., 2018). All of these analyses were implemented in R 4.3.1 using the TwoSampleMR R package (Hemani et al., 2018).

## Results

### Overview of the study

**Figure 1.**
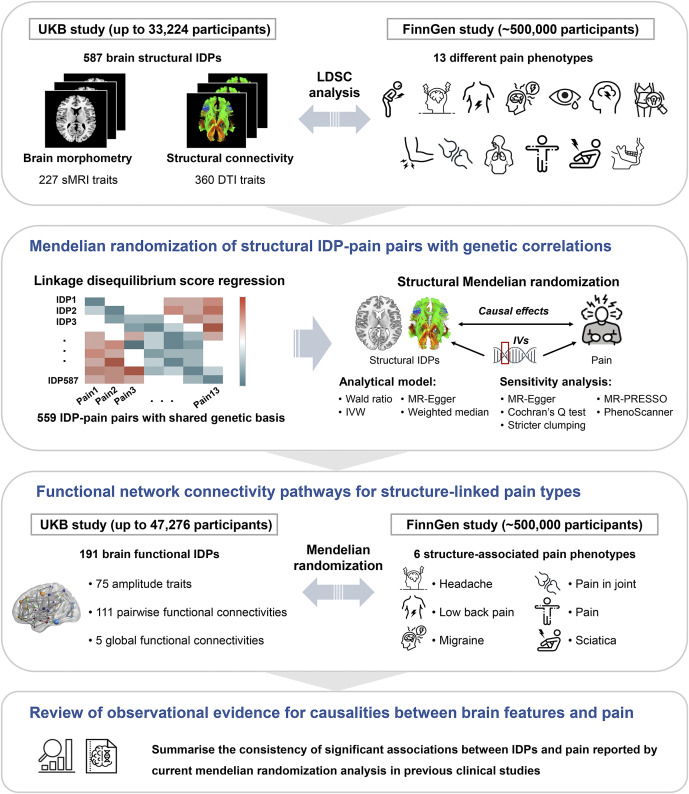
Illustration of the study design. Abbreviations: UKB, UK Biobank; IDP, brain imaging-derived phenotype; sMRI, structural magnetic resonance imaging; DTI, diffusion tensor imaging; LDSC, linkage disequilibrium score; IV, instrumental variable; IVW, inverse variance weighted; MR, Mendelian randomization. Figure 1. long description.

**Figure 2.**
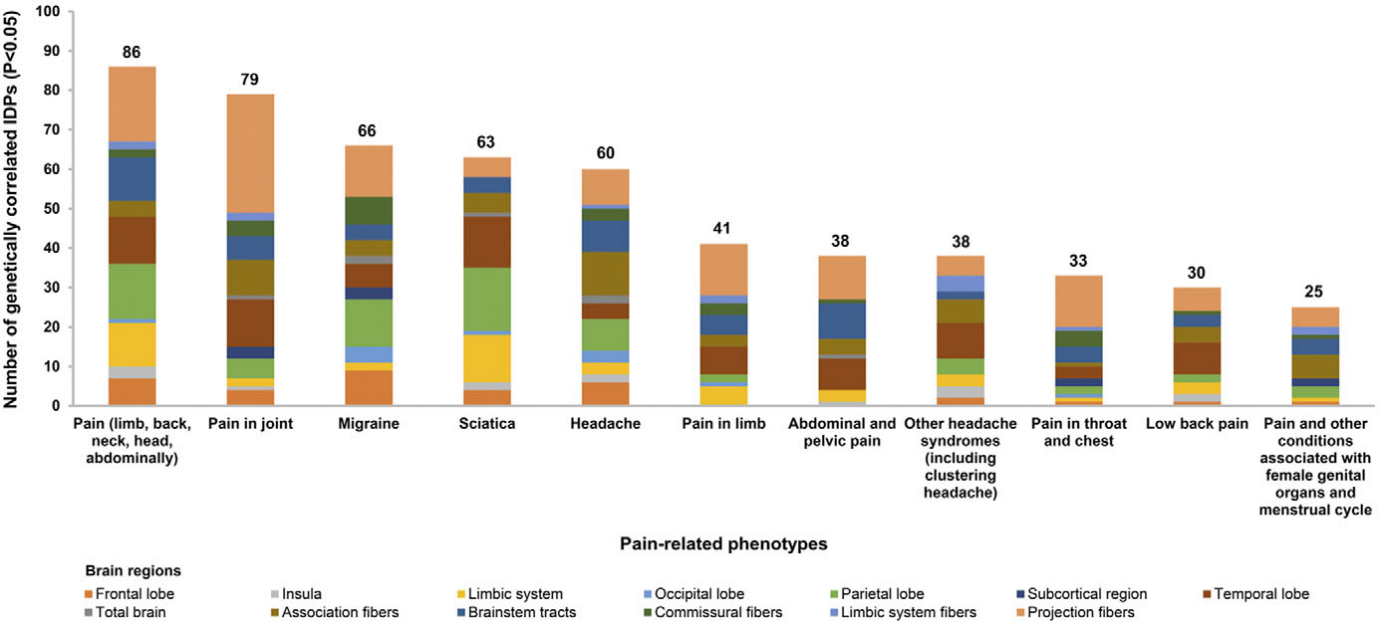
The genetic correlation between brain structure and pain. Figure 2. long description.

The study design is illustrated in Figure 1. We first selected 587 reliably measured brain structural IDPs from a sample size of up to 33,224 UK Biobank participants (Smith et al., 2021). These measurements involved the cerebral cortex, subcortical regions, and WM tracts. GWAS summary data for 13 pain-related phenotypes from the FinnGen study were collected, which were conducted among approximately 500,000 participants from Finland (Kurki et al., 2023). We examined the pairwise genetic correlations between the 587 structural IDPs and the 13 pain-related phenotypes and found that 559 structural IDP–pain pairs with a nominal *P*-value of less than 0.05 (Figure 2 and Supplementary Table S4). We then conducted a bidirectional two-sample MR analysis to further examine the causality of these identified pairs of brain structural changes and pain. The MR analyses suggested a total of 20 pairs of significant causal associations for six different pain-related phenotypes in both forward and reverse analyses. For those six pain-related phenotypes, we further conducted MR analyses to examine their associations with 191 functional IDPs and identified 22 significant associations. Lastly, we summarized available corresponding observational evidence for identified associations between IDPs and pain-related phenotypes.

### LDSC regression analysis

We used LDSC to evaluate the genetic correlations between 587 structural IDPs and 13 pain-related phenotypes, of which 559 IDP–pain pairs displayed nominally significant genetic correlations (*P* < 0.05) (Figure 2). These included 11 specific types of pain, with correlation coefficients ranging from −0.26 to 0.28. However, no genetic correlation was found for ocular pain and TMD muscular pain linked with fibromyalgia. These pairs with significant genetic correlations were further analyzed through bidirectional MR analysis. Supplementary Table S4 provides the detailed results of these genetic correlations.

### IV selection for MR analysis

After clumping SNPs by linkage disequilibrium and replacing absent SNPs with their proxies, the remaining SNPs that served as IVs for structural IDPs and pain-related phenotypes in the bidirectional MR analysis are presented in Supplementary Table S5. Instrument strength was evaluated using F-statistics, all of which exceeded 10, indicating that these SNPs are appropriate IVs and not weak instruments.

### Pain-related phenotypes and structural IDPs

**Figure 3.**
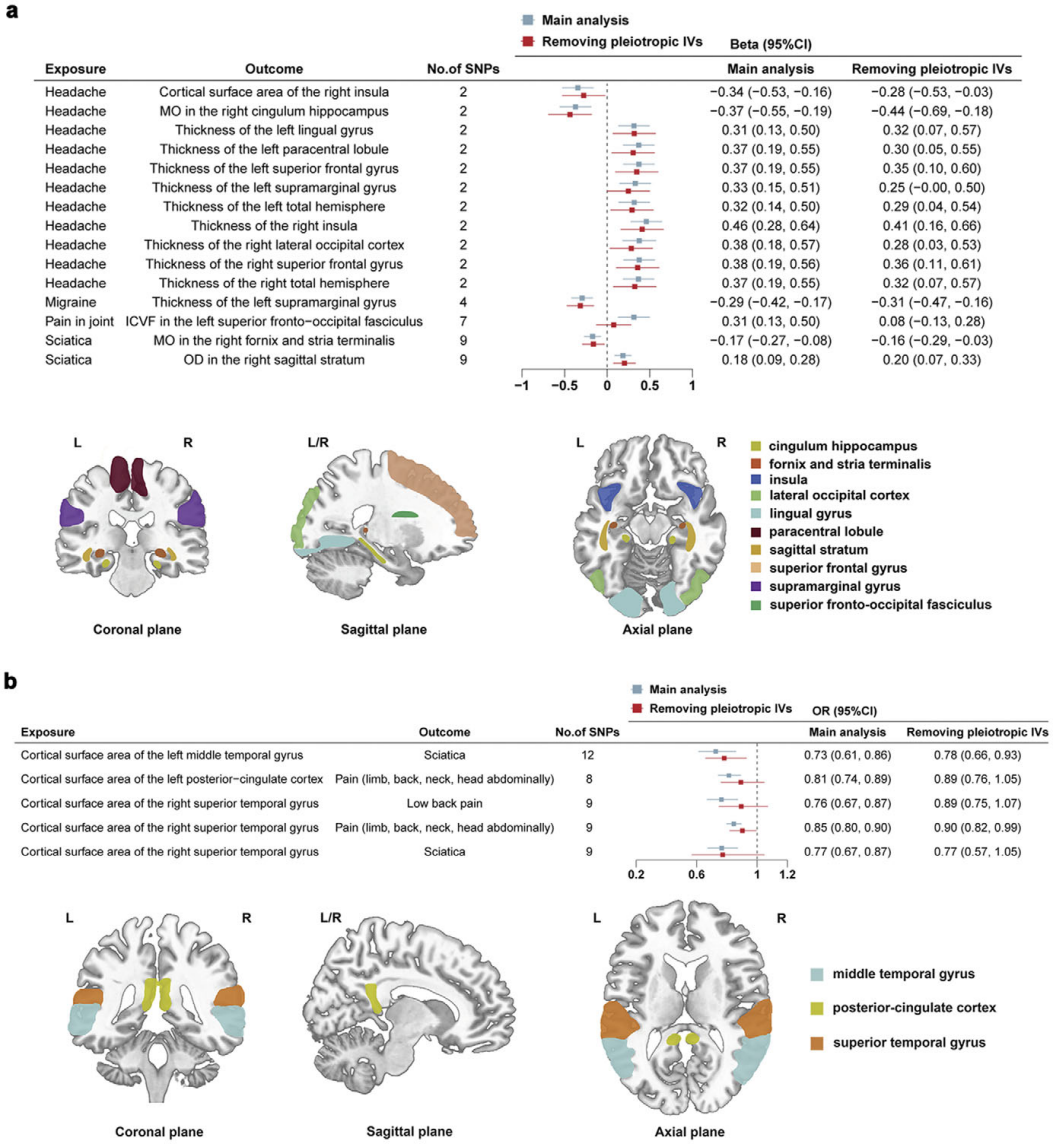
The bidirectional MR analysis results for the effects of pain-related phenotypes and structural IDPs with FDR-adjusted *P*-values < 0.05. The forest plot shows significant MR results based on the FDR-adjusted *P*-value estimated from either the inverse variance weighted method or the Wald ratio method from 1,107 tests conducted in both directions. The main analysis included all IVs retained from the primary IV selection procedures. In the analysis removing pleiotropic IVs, the IVs associated with possible confounders such as smoking, drinking, socioeconomic status, education, and various psychiatric disorders in the PhenoScanner database of *P*-value <1 × 10^−5^ were excluded. The bottom panel shows the anatomical regions of the brain for the corresponding IDPs bilaterally. Brain regions were overlaid on the ch2better template using Anatomical Automatic Labeling (AAL) or JHU DTI-based white-matter atlases in MRIcron software v1.0 (https://www.nitrc.org/projects/mricron/). Figure 3. long description.

In bidirectional MR analysis, we identified significant effects in 15 pairs where genetically predicted risks for four pain-related phenotypes – headache, migraine, joint pain, and sciatica – were causally associated with structural IDPs, and in five pairs where changes in cortical surface area (per one standard deviation increase) affected three pain phenotypes after FDR correction (Figure 3).

Headache was associated with 11 IDPs, including increased cortical thickness in nine regions involved in pain perception and emotional processing: the left lingual gyrus, paracentral lobule, superior frontal gyrus (bilateral), supramarginal gyrus, right insula, lateral occipital cortex, and total hemisphere (IVW *β* = 0.31 to 0.46, FDR-corrected *P* < 0.05) (Figure 3a and Supplementary Tables S6 and S7). Additionally, headache was linked to decreased surface area in the right insula (IVW *β* = −0.34, 95% CI = −0.53 to −0.16, *P* = 1.97 × 10^−4^) and MO in the right cingulum hippocampus (IVW *β* = −0.37, 95% CI = −0.55 to −0.19, *P* = 6.83 × 10^−5^). Although multiple associations between headache and structural IDPs were identified in the European dataset, these findings were not replicated in the Asian population, with effect estimates generally attenuated and lacking statistical significance, suggesting the possibility of population-specific effects (Supplementary Table S8). Migraine, a primary headache disorder with associated visual and sensory symptoms, showed a negative association with thickness in the left supramarginal gyrus, a region related to pain perception and emotional reactivity (IVW *β* = −0.29, 95% CI = = − 0.42 to −0.17, *P* = 2.72 × 10^−6^). Joint pain was positively associated with ICVF in the left superior fronto-occipital fasciculus (IVW *β* = 0.31, 95% CI = 0.13–0.50, *P* = 8.29 × 10^−4^), a pathway connecting the occipital, parietal, and superior frontal lobes. Sciatica, a symptom of nerve-related pain radiating to the leg, was linked to decreased MO in the right fornix and stria terminalis (IVW *β* = −0.17, 95% CI = −0.27 to −0.08, *P* = 4.53 × 10^−4^) and increased OD in the right sagittal stratum (IVW *β* = 0.18, 95% CI = 0.09–0.28, *P* = 1.63 × 10^−4^), regions involved in sensory, motor, and emotional processing.

In reverse MR analyses, five significant effects linked genetically predicted increases in cortical surface area to attenuated pain phenotypes after FDR correction (Figure 3b and Supplementary Tables S9 and S10). For example, increased surface area in the left middle temporal and posterior cingulate cortices was associated with lower risks of sciatica (IVW OR = 0.73, 95% CI = 0.61–0.86, *P* = 1.91 × 10^−4^) and overall pain (IVW OR = 0.81, 95% CI = 0.74–0.89, *P* = 7.36 × 10^−6^), respectively. Similarly, increased surface area in the right superior temporal gyrus correlated with lower risks of low back pain (IVW OR = 0.76, 95% CI = 0.67–0.87, *P* = 6.60 × 10^−5^), overall pain (IVW OR = 0.85, 95% CI = 0.80–0.90, *P* = 7.35 × 10^−9^), and sciatica (IVW OR = 0.77, 95% CI = 0.67–0.87, *P* = 5.25 × 10^−5^). After adjusting for potential confounders in multivariable MR analyses, the associations between genetically proxied structural IDPs and pain remained statistically significant and directionally consistent (IVW OR = 0.67 to 0.95), indicating robustness of the observed effects (Supplementary Table S11).

### Pain-related phenotypes and functional IDPs

**Figure 4.**
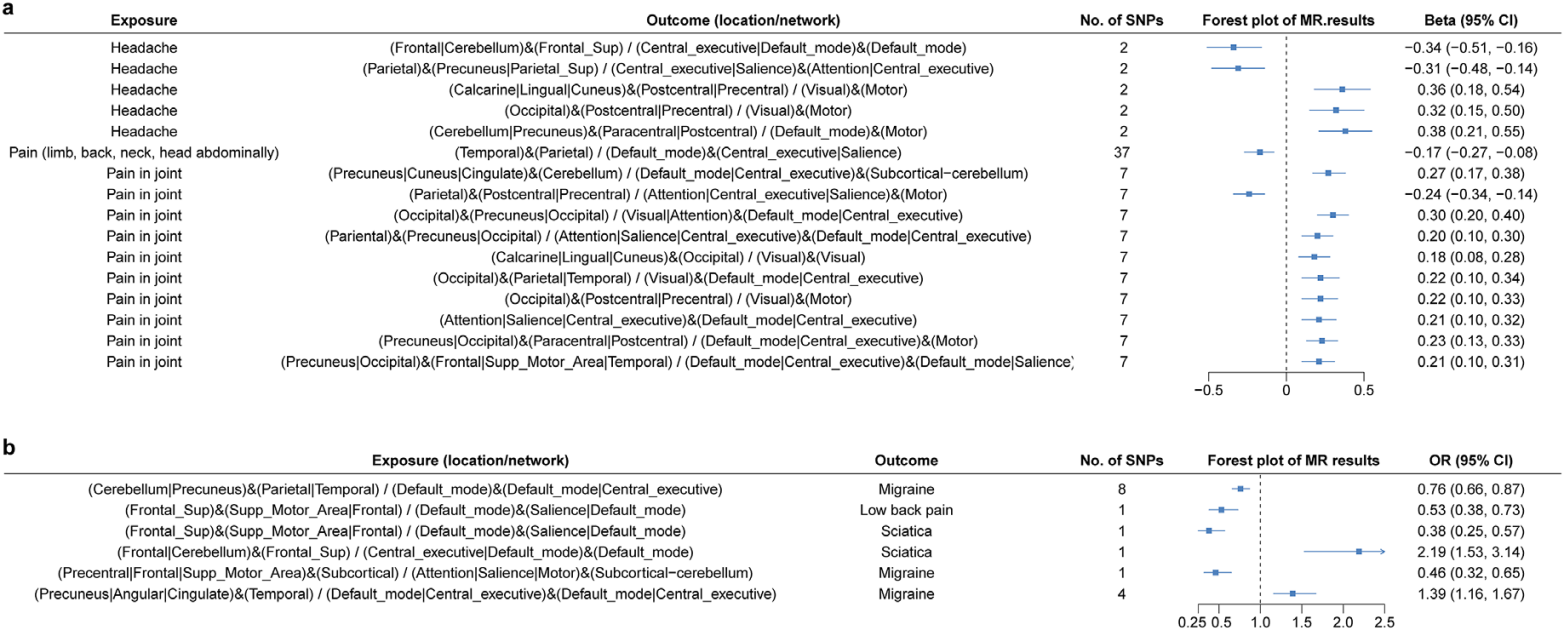
The bidirectional MR analysis results for the effects of pain-related phenotypes and functional IDPs with FDR-adjusted *P*-values < 0.05. The forest plot shows significant MR results based on the FDR-adjusted *P*-value estimated from either the inverse variance weighted method or the Wald ratio method from 2,292 tests conducted in both directions. (a) Effects of pain-related phenotypes on functional IDPs. (b) Effects of functional IDPs on pain-related phenotypes. Figure 4. long description.

For the six pain-related phenotypes associated with structural changes, we identified 16 causal effects from pain to functional IDPs, linking headache, joint pain, and overall pain with various functional connectivity networks (Figure 4a and Supplementary Table S12). Pain broadly influenced networks such as the default network, central executive network, salience, attention, visual, motor, and subcortical cerebellum. Specifically, headache was significantly associated with connectivity in regions such as the cerebellum, parietal lobe, and precentral/postcentral gyri (*β* = −0.34 to 0.38), suggesting its impact on cognitive and emotional responses to pain. Joint pain affected connections to emotion- and perception-related networks involving the precuneus, parietal, and occipital lobes (*β* = −0.24 to 0.30). Overall pain negatively affected connectivity in the temporal gyrus and parietal regions (IVW *β* = −0.17, 95% CI: −0.27 to −0.08, *P* = 4.48 × 10^−4^). Notably, the default mode and central executive networks were broadly affected by these pain phenotypes, suggesting a generalized role in pain modulation. All inferred estimates by other models are presented in Supplementary Table S12.

Genetically predicted connectivity changes also exhibited significant effects on pain phenotypes, notably migraine, sciatica, and low back pain (Figure 4b and Supplementary Table S13). Two functional IDPs involving the default mode and central executive networks were linked to migraine, with connectivity spanning the precuneus, angular gyrus, cingulate gyrus, and temporal lobe (IVW OR = 1.39, 95% CI = 1.16–1.67, *P* = 4.77 × 10^−4^), as well as connectivity in the cerebellum and parietal regions (IVW OR = 0.76, 95% CI = 0.66–0.87, *P* = 1.46 × 10^−4^). Besides, connectivity in precentral, frontal, and subcortical areas, which affect networks including attention, salience, motor, and subcortical cerebellum, was associated with decreased migraine risk (IVW OR = 0.46, 95% CI = 0.32–0.65, *P* = 1.87 × 10^−5^). Neural activity between the frontal gyrus/cerebellum and superior frontal gyrus, involving functional connectivity of the central executive and default mode networks, was positively associated with sciatica (IVW OR = 2.19, 95% CI: 1.53–3.14, *P* = 2.11 × 10^−5^), while spontaneous activity in the superior frontal gyrus and supplementary motor area was negatively associated with low back pain (IVW OR = 0.53, 95% CI: 0.38–0.73, *P* = 1.61 × 10^−4^) and sciatica (OR = 0.38, 95% CI: 0.25–0.57, *P* = 3.70 × 10^−6^) (Figure 4b and Supplementary Table S13).

### Sensitivity analyses

The estimates from the weighted median and MR-Egger methods aligned consistently with the primary IVW results (Supplementary Tables S6 and S9). All significant pairs in the MR passed Cochran’s Q and MR-Egger intercept tests (*P* > 0.05), indicating no significant horizontal pleiotropy (Supplementary Tables S6 and S9). MR-PRESSO test identified one potential outlier for the effect of joint pain on ICVF in the left superior fronto-occipital fasciculus, but after removing it, the estimates remained consistent with the primary results (Supplementary Table S6). Applying a stricter 10 Mb clumping window also yielded results consistent with the primary analysis, despite fewer SNPs being used (Supplementary Figures S1 and S2). Sensitivity analysis excluding IVs related to potential confounders showed the same directions as the primary results, though with wider CIs due to fewer IVs (Figure 3 and Supplementary Tables S7 and S10). Overall, these sensitivity analyses supported the robustness of the identified associations.

### Observational evidence of the MR findings

Table 1 provides a summary of previous literature on associations between IDPs and pain-related phenotypes, supporting our findings. Out of 1702 studies identified in PubMed, 520 unique studies were screened, and 15 met the inclusion criteria. Prior research, primarily observational, has focused on associations between cortical thickness and surface area with pain phenotypes, with no studies on IDPs like MO, ICVF, or OD. Most studies examined headache, migraine, and sciatica, with limited brain imaging research on other pain types. Participants included both genders across a wide age range, with sample sizes from 26 to 819. Observed associations in these studies generally show directional consistency with our MR findings. Although fMRI is commonly reported in pain research, reproducibility issues of modality results (e.g. predefined hypotheses, data accessibility, and region of interest-based analysis) limit the comparability with our MR findings of functional connectivity; thus, we focused on structural-related associations for consistency (Botvinik-Nezer et al., 2020).

**Table 1.** Literature review of observational evidence relating to current MR findings Table 1. long description.

Pain	IDPs	MR effect^a^	Ref. (PMID)	Sample size	Conclusion
Headache	Thickness of LING.L	(+)	37144161	35 migraineurs (20 with exclusively visual aura, 15 with complex neurological aura), 19 HCs	Migraineurs with exclusively visual aura < HCs
			29360944	166 female patients with migraine with aura, 137 HCs	Female patients > HCs
Headache	Thickness of PCL.L	(+)	29809228	Seven patients with migraine with aura and 12 patients with migraine without aura, 19 HCs	Patients > HCs
Headache	Thickness of SFG.L	(+)	35861130	13 NDPH patients, 13 age- and sex-matched HCs	NDPH patients < controls
			37144161	20 migraineurs with exclusively visual aura, 15 migraine patients with complex neurological aura, 19 HCs	Patients of migraine with complex neurological aura < HCs
			28831776	28 patients with persistent PTH, 28 individuals with migraine, 28 HCs	PTH patients < HCs
			29139130	33 patients with PTH, 33 HCs	PTH patients < HCs
			41652332	26 patients with episodic cluster headache, 20 HCs	Cluster headache patients < HCs
Headache	Thickness of SMG.L	(+)	30525946	131 migraineurs (38 with and 93 without aura), 115 HCs	Patients without aura < HCs
			23533286	63 migraine patients (32 with and 31 without aura), 18 HCs	Migraine patients with aura > HCs and migraine patients without aura
			34294048	72 pediatric patients affected by migraine without aura, 82 HCs	Patients < HCs
Headache	Thickness of left total hemisphere	(+)	24812034	20 patients with aura symptoms, 13 patients with regard to headache	Headache side < non-headache side
Headache	Thickness of INS.R	(+)	28733941	32 migraineurs without aura, 32 HCs	Migraineurs < HCs
			37144161	20 migraineurs with exclusively visual aura, 15 migraineurs with complex neurological aura, 19 HCs	Migraineurs with exclusively visual aura < HCs
			23533286	63 migraineurs, 18 HCs	Migraineurs with aura < HCs and migraine patients without aura
Headache	Thickness of SFG.R	(+)	37144161	20 migraineurs with exclusively visual aura, 15 migraineurs with complex neurological aura, 19 HCs	Migraineurs with exclusively visual aura < HCs
			23533286	63 migraine patients (24 with and 39 without WMHs), 18 HCs	Patients with WMHs < HCs
			41168693	415 children with multisite pain, 404 HCs	Patients with multisite pain < HCs
Headache	Thickness of right total hemisphere	(+)	24812034	20 patients with aura symptoms, 13 patients with regard to headache	Headache side < non-headache side
Headache	Thickness of SMG.L	(−)	34294048	72 pediatric migraineurs without aura, 82 HCs	Patients < HCs
			30525946	131 migraineurs (38 with and 93 without aura), 115 HCs	Patients without aura < HCs
			23533286	63 migraine patients (32 with and 31 without aura), 18 HCs	Migraineurs with aura > HCs
Pain	Cortical surface area of STG.R	(−)	33040149	57 episodic migraineurs, 57 chronic migraineurs, and 52 HCs	Chronic migraineurs < HCs
			23533286	63 migraine patients (32 with and 31 without aura), 18 HCs	Migraineurs > HCs
Sciatica	Cortical surface area of STG.R	(−)	36532276	34 chronic sciatica patients, 36 HCs	Chronic sciatica patients > HCs

a The notations ‘+’ and ‘–’ refer to positive and negative causal associations in MR analyses.

Abbreviations: LING.L, left lingual gyrus; HC, healthy controls; PCL.L, left paracentral lobule; SFG.L, left superior frontal gyrus; NDPH, new daily persistent headache; PTH, post-traumatic headache; SMG.L, left supramarginal gyrus; INS.R, right insula; SFG.R, right superior frontal gyrus; WMH, white matter hyperintensity; STG.R, right superior temporal gyrus.

## Discussion

In this study, we integrated structural and functional perspectives to comprehensively examine the genetic relationships between IDPs and pain-related phenotypes. We began by identifying genetic correlations between 559 pairs of structural IDPs and pain-related phenotypes. Additional evidence from bidirectional MR analyses supported potential causal relationships between brain structural and functional alterations and pain. These findings underscore the value of multimodal neuroimaging as potential biomarkers to inform target regions and neurobiological mechanisms in clinical contexts.

In the forward structural MR analysis of pain on structural IDPs, we identified significant associations between headache, migraine, joint pain, sciatica, and structural alterations in multiple brain regions. Previous observational studies have shown significant structural differences between pain patients and healthy controls, with chronic pain linked to reductions in cortical and subcortical GM areas associated with pain processing (Gustin et al., 2011; Winkler et al., 2018). Among the pain phenotypes analyzed, headache exhibited the most consistent associations, particularly with higher cortical thickness in several brain regions, aligned with previous observational studies of changes in the left lingual gyrus, left paracentral lobule, and left supramarginal gyrus (Amaral et al., 2018; Gaist et al., 2018; Messina et al., 2013). Some studies also indicated that there is a significant thinning of the left hemisphere (lingual gyrus, superior frontal gyrus, supramarginal gyrus) and the right hemisphere (insula and superior frontal gyrus) in patients with headaches (Abagnale et al., 2023; Chong et al., 2018; Guarnera et al., 2021; Magon et al., 2019; Schwedt et al., 2017; Szabo et al., 2022; J. Zhang et al., 2017). A feasible explanation is that MR captures the effect of lifelong genetic predisposition, whereas observational studies typically measure brain structure after disease onset and progression, capturing downstream consequences of chronic pain (Swanson, Tiemeier, Ikram, & Hernan, 2017). The increased cortical thickness observed in our MR analysis may represent a predisposing neuroanatomical feature, potentially reflecting heightened sensory sensitivity, increased synaptic or dendritic complexity, or altered neurodevelopmental trajectories in pain-related regions. In contrast, cortical thinning observed in patients may arise as a consequence of prolonged pain exposure, neuroplastic changes, or stress-related neurodegeneration (Chong, Dodick, Schlaggar, & Schwedt, 2014). These findings also suggest that headache attacks may induce progressive structural changes in key areas involved in pain perception (lingual gyrus and insula), pain modulation (insula), and cognitive functions (superior frontal gyrus and supramarginal gyrus) (Biggs et al., 2020; Khera & Rangasamy, 2021; la Fougere et al., 2011; May, 2008; Zhang et al., 2020). As a clinically defined subtype of headache, migraine demonstrated fewer significant associations compared to the broader headache phenotype. The broader headache category likely captures shared genetic liability across recurrent headache disorders, potentially explaining the generalized associations observed (Headache Classification Committee of the International Headache Society (IHS), The International Classification of Headache Disorders, 3rd edition, 2018). Migraine was uniquely associated with decreased cortical thickness in the left supramarginal gyrus, consistent with prior evidence indicating cortical thinning in individuals (Magon et al., 2019; Zharova et al., 2026). However, cortical thickness changes during the headache phase of migraine suggest dynamic, reversible cortical alterations in pain-related areas. The observed decrease in cortical thickness on the side of pain during attacks, accompanied by increased cortical thickness in the contralateral hemisphere, highlights the importance of interpreting these findings in the context of the complex pathophysiological and clinical characteristics (Chaudhry et al., 2025). Beyond GM morphology, pain phenotypes also influenced WM microstructure. Joint pain was linked to higher ICVF in the left superior fronto-occipital fasciculus, a region integral to sensory processing and motor control, suggesting increased myelinated neurite density and intracellular water content (Tu, Wang, Xiong, & Gao, 2022; Zhang, Schneider, Wheeler-Kingshott, & Alexander, 2012). Sciatica was positively associated with OD in the right sagittal stratum, indicative of higher dendritic and axonal complexity (Mioduszewski et al., 2020; Zhang et al., 2012).

In the reverse structural MR analysis, we identified negative associations between increased surface area in the left middle temporal gyrus, left posterior cingulate cortex, and right superior temporal gyrus with sciatica, low back pain, and overall pain. Importantly, after adjusting for a range of potential confounders in multivariable MR analyses, the associations remained statistically significant and directionally consistent, supporting the notion that these effects are not driven by shared genetic influences and may represent independent causal contributions of structural IDPs to pain. Surface area, which reflects the density of cortical columns, may serve as a sensitive metric for disease-related changes (Christensen et al., 2024). These regions play critical roles in pain processing and regulation, with their decreased surface area potentially indicating suppression. Specifically, the superior and middle temporal gyri are implicated in sensory integration, the middle temporal gyrus in expectation localization, and the posterior cingulate cortex in nonemotional responses to harmful stimuli (Goossens et al., 2018; Lin, Lee, & Weng, 2016; Wei et al., 2022). Overall, bidirectional interactions between structural IDPs and pain suggest distinct underlying mechanisms, where cortical surface features reflect susceptibility to specific pain phenotypes, whereas pain-related structural alterations represent potential targets for intervention.

Structural traits primarily reflected relatively stable neuroanatomical alterations, such as changes in cortical thickness and regional morphology, which may represent long-term vulnerability and biomarkers of pain-related processes. In contrast, functional IDPs capture dynamic network-level processes of brain activity and connectivity, reflecting more flexible and state-dependent neural responses (Parsaei, Taebi, Arvin, & Moghaddam, 2024). This structural–functional coupling supports the notion that chronic or recurrent pain conditions may arise from the interaction between pre-existing anatomical predispositions and ongoing functional reorganization. Our functional analyses identified significant associations with three core cognitive networks – the salience network, central executive network, and default mode network – emphasizing their critical roles in pain processing (Menon, 2011). These networks are not independent modules but dynamically interact to regulate attention, self-referential processing, and executive control in response to salient stimuli such as pain. The salience network, responsible for detecting salient stimuli such as pain, reallocates attentional resources from the default mode network to the central executive network, enabling more effective responses to pain (De Ridder, Vanneste, Smith, & Adhia, 2022). These networks further interact with higher-order systems that mediate the cognitive, emotional, and contextual dimensions of pain processing. According to network science, different dimensions of pain are mediated by specific connectivity patterns within resting-state networks: the salience network (suffering), default mode network (self-representation), central executive network (cognitive processing), attention and visual networks (context and focus), motor network (coping responses), and subcortical-cerebellar systems (emotion and control) (De Ridder et al., 2022). This interconnected network framework provides a holistic explanation for the multifaceted pain experience, spanning sensory perception, emotional regulation, and behavioral responses and aligns with the MR associations observed between functional network changes and pain phenotypes in our study.

A key finding of this study is the identification of three brain regions – the superior frontal gyrus, lingual gyrus, and paracentral lobule – in headache, as reflected by both structural differences and alterations in functional connectivity. Headache was associated with increased cortical thickness across all three regions, accompanied by distinct patterns of neural activity: lower activity in the superior frontal gyrus and increased activity in both the lingual gyrus and paracentral lobule. As a key region of the default mode network, the superior frontal gyrus plays an essential role in self-regulation, adapting behavior in response to pain, and eliciting positive emotions (Briggs et al., 2020). Meanwhile, the projections from the superior frontal gyrus to the thalamus and periaqueductal gray constitute the descending inhibitory pain control system and participate in the modulation of autonomic responses to pain (Dirupo et al., 2025). The dissociation of increased cortical thickness and reduced intrinsic activity in this region may reflect a compensatory structural adaptation in response to persistent nociceptive input, coupled with functional suppression due to maladaptive network regulation. Consistent with these findings, previous studies have demonstrated decreased functional activity in this region among individuals with medication-overuse headache and migraine without aura (Feng et al., 2022; Li et al., 2023). In contrast, the lingual gyrus, a key component of the visual network, showed both increased cortical thickness and elevated intrinsic activity. Previous evidence has linked this region to emotional regulation of nociception and situational processing (Cui et al., 2021; Wen et al., 2023). The concurrent structural and functional enhancement observed here may indicate experience-dependent plasticity driven by repeated nociceptive stimulation, particularly given the well-established link between visual processing abnormalities and headache disorders such as migraine. Enhanced activity in the lingual gyrus may therefore represent an adaptive or sensitized response to persistent sensory input. Similarly, the paracentral lobule, part of the sensorimotor network, demonstrated increased cortical thickness alongside heightened intrinsic activity. This region serves as a crucial part of the sensorimotor network and is centrally involved in pain perception and sensorimotor integration (Kim, Kim, & Nabekura, 2017; Wei et al., 2022). Previous studies have indicated that its activity is elevated during migraine attacks and positively correlates with attack frequency (Joppekova et al., 2025). In addition, the activation of the paracentral lobule will affect the connections between the sensorimotor network and other networks, which may influence the subsequent outcome of migraines, as well as whether it will develop into a chronic migraine and the remodeling of the brain (Lei & Zhang, 2021; Liu et al., 2021). Collectively, these findings suggest that structural and functional alterations in headache are not uniformly aligned but instead exhibit region-specific patterns of coupling and dissociation. This highlights a complex interplay in which structural remodeling interacts with dynamic network activity to shape the overall neural phenotype of pain.

This study is the first to establish genetic correlations and causal relationships between brain structural and functional IDPs and pain, using the largest neuroimaging genetics dataset currently available. A major strength of this study lies in the application of MR, which offers genetically inferred associations by effectively reducing confounding factors compared to traditional cross-sectional studies in this field. Another strength is the inclusion of diverse multimodal neuroimaging phenotypes and various pain types, providing a comprehensive view of how brain structures, neural activity, and functional connectivity contribute to pain risk.

This study also has some limitations. First, while the FinnGen consortium leverages the advantage of large sample sizes by defining pain phenotypes through clinical diagnoses, it must be acknowledged that reported symptoms may introduce potential inaccuracies. In particular, ICD-based definitions do not explicitly distinguish between acute and chronic pain, potentially encompassing both transient and persistent manifestations. Moreover, the broad headache phenotype may include multiple subtypes, including migraine, which could not be explicitly separated in the summary-level data, limiting the ability to determine whether specific headache subtypes drive the observed associations. Therefore, our findings should be interpreted as reflecting genetic relationships with clinically ascertained pain conditions. Second, the main analysis restricted its included samples to individuals of European ancestry only, and the limited evidence related to headaches suggests differences among different ethnicities. Therefore, future multiancestry genetic studies will be critical for cross-population discovery and replication. Third, MR results reflect the lifetime effects of exposure factors on outcome traits. This is not equivalent to an exposure occurring at a specific point in time or over a short period (Swanson et al., 2017). Therefore, the estimated MR effect sizes should be interpreted with caution and not treated as the effect of traditional real-world exposure. Moreover, the limited number of IVs for some IDPs may have constrained the statistical power underlying causal inference, and future large-scale GWAS are warranted to identify additional instruments and further strengthen causal analyses. Lastly, given the weaker genetic effects on functional brain networks than on structures, this part of the MR analysis was conducted as a complementary exploration on top of the structural IDP results. Therefore, further clinical evaluation with large-scale multimodal neuroimaging studies and associated genetic statistics is warranted for validation.

## Conclusions

In conclusion, our study comprehensively examined the genetically inferred associations between numerous brain structural and functional imaging features and multiple pain presentations using MR analysis. The identified associations between brain imaging traits and pain phenotypes highlight the complex neurobiological basis of pain-related conditions. These findings add new perspectives on the potential links among brain structure, function, and pain, potentially supporting future investigations into the neurological mechanisms of pain.

## Supporting information

10.1017/S0033291726105091.sm001 Mao et al. supplementary material Mao et al. supplementary material

## Data Availability

The datasets supporting the conclusions of this article are publicly available. Summary statistics for structural IDPs were publicly available from the Oxford Brain Imaging Genetics (BIG40) web browser (https://open.win.ox.ac.uk/ukbiobank/big40/). Summary statistics for functional IDPs can be obtained via the Zenodo platform (https://zenodo.org/records/5775047). Summary statistics for pain phenotypes were obtained from the FinnGen Consortium and can be downloaded from https://www.finngen.fi/en/access_results. Summary statistics for multivariable MR analysis were available from the OpenGWAS project (GWAS ID: ukb-b-7408, ebi-a-GCST90029028, ebi-a-GCST90029013, ieu-a-1283, ukb-b-19953, ieu-b-4877, and ukb-b-18336). Summary statistics for replication analysis between headache and structural IDPs in the Asian population can be obtained from the OpenGWAS project (GWAS ID: ukb-e-3799_CSA) and the GWAS Catalog (https://www.ebi.ac.uk/gwas/publications/38811844). All data needed to evaluate the conclusions in the paper are present in the paper and/or the Supplementary Material.

## Supplementary material

The supplementary material for this article can be found at http://doi.org/10.1017/S0033291726105091.

## Long descriptions

### Figure 1. Long description

The flowchart is organized into four sequential horizontal panels.

Panel 1: Discovery of genetic correlations. On the left, UKB study data includes up to 33,224 participants and 587 brain structural IDP s, categorized into brain morphometry (227 sMRI traits) and structural connectivity (360 DTI traits). On the right, FinnGen study data includes approximately 500,000 participants and 13 different pain phenotypes represented by anatomical icons. A double-headed arrow labeled LDSC analysis connects the two datasets.

Panel 2: Mendelian randomization of structural IDP-pain pairs. A heatmap on the left shows Llinkage disequilibrium score regression for 559 IDP-pain pairs with a shared genetic basis. An arrow points right to a structural Mendelian randomization diagram. This diagram shows causal effects between structural IDPs and pain, mediated by IVs (represented by DNA helices). Analytical models listed include Wald ratio, IV W, MR-Egger, and Weighted median. Sensitivity analyses listed include MR-Egger, Cochran's Q test, Stricter clumping, MR-PRESS O, and PhenoScanner.

Panel 3: Functional network connectivity pathways. On the left, UKB study data for 47,276 participants includes 191 brain functional IDPs (75 amplitude traits, 111 pairwise functional connectivities, and 5 global functional connectivities). On the right, FinnGen study data includes 6 structure-associated pain phenotypes: Headache, Low back pain, Migraine, Pain in joint, Pain, and Sciatica. A double-headed arrow labeled Mendelian randomization connects them.

Panel 4: Review of observational evidence. This final section summarizes the consistency of significant associations between IDPs and pain reported by current MR analysis in previous clinical studies, accompanied by icons of a magnifying glass over a chart and a document.

Navigate back to Figure 1..

### Figure 2. Long description

The y-axis is labeled Number of genetically correlated IDPs (P < 0.05) with a scale from 0 to 100. The x-axis is labeled Pain-related phenotypes. A legend at the bottom identifies 13 brain regions represented by different colors within the stacked bars: Frontal lobe, Insula, Limbic system, Occipital lobe, Parietal lobe, Subcortical region, Temporal lobe, Total brain, Association fibers, Brainstem tracts, Commissural fibers, Limbic system fibers, and Projection fibers.

The bars are arranged in descending order of total IDP count from left to right:

* Pain (limb, back, neck, head, abdominally): 86

* Pain in joint: 79

* Migraine: 66

* Sciatica: 63

* Headache: 60

* Pain in limb: 41

* Abdominal and pelvic pain: 38

* Other headache syndromes (including clustering headache): 38

* Pain in throat and chest: 33

* Low back pain: 30

* Pain and other conditions associated with female genital organs and menstrual cycle: 25

In all bars, Projection fibers (light orange) and Association fibers (olive green) consistently represent the largest segments of the total count.

Navigate back to Figure 2..

### Figure 3. Long description

Panel a displays a forest plot with columns for Exposure, Outcome, Number of SNPs, a Beta coefficient plot with 95 percent C I, and numerical values for Main analysis and Removing pleiotropic IVs. Exposures include Headache, Migraine, Pain in joint, and Sciatica. Outcomes involve cortical surface area, thickness, M O, ICV F, and OD of various brain regions. The Beta values range from minus 1 to 1, with a vertical dashed line at 0. Below the plot, three brain cross-sections (Coronal, Sagittal, and Axial planes) highlight regions like the cingulum hippocampus, insula, and supramarginal gyrus in various colors corresponding to a legend on the right.

Panel b follows a similar structure but focuses on brain structural IDPs as exposures and pain phenotypes as outcomes. Exposures include cortical surface area of the left middle temporal gyrus, left posterior-cingulate cortex, and right superior temporal gyrus. Outcomes include Sciatica, Low back pain, and general pain. The plot shows Odds Ratios (O R) ranging from 0.2 to 1.2, with a vertical dashed line at 1. The anatomical maps below highlight the middle temporal gyrus, posterior-cingulate cortex, and superior temporal gyrus in light blue, yellow-green, and orange respectively.

Navigate back to Figure 3..

### Figure 4. Long description

The image consists of two horizontal panels, a and b.

Panel a displays the effects of pain-related phenotypes as the Exposure on functional IDPs as the Outcome. It lists 16 rows. The first five rows use Headache as the exposure, followed by one row for general pain and 10 rows for Pain in joint. The forest plot shows data points with horizontal error bars relative to a vertical dashed line at zero. Significant results for Headache show negative beta values around minus 0.3 and positive values around 0.35. Pain in joint results are mostly positive, clustered between 0.18 and 0.30, except for one negative result at minus 0.24. The number of SNPs ranges from 2 to 37.

Panel b displays the effects of functional IDPs as the Exposure on pain-related phenotypes as the Outcome. It lists 6 rows. The outcomes include Migraine, Low back pain, and Sciatica. The forest plot is measured against a vertical dashed line at 1.0 for Odds Ratio. Four results are below 1.0, indicating a protective effect, with OR values ranging from 0.38 to 0.76. Two results are above 1.0, including a significant effect on Sciatica with an OR of 2.19 and Migraine with an OR of 1.39. The number of SNPs for these tests ranges from 1 to 8.

Navigate back to Figure 4..

### Table 1. Long description

A table with six columns: Pain, IDPs, MR effect, Ref. (P MID), Sample size, and Conclusion.

* Headache and Thickness of LING dot L: Positive MR effect. One study shows migraineurs with visual aura have lower thickness than HCs, while another shows female patients have higher thickness than H Cs.

* Headache and Thickness of PCL dot L: Positive MR effect. Patients with migraine with or without aura have higher thickness than H Cs.

* Headache and Thickness of SFG dot L: Positive MR effect. Four studies indicate NDP H, migraine with complex aura, PT H, and cluster headache patients all have lower thickness than H Cs.

* Headache and Thickness of SMG dot L: Positive MR effect. Studies show patients without aura have lower thickness than HCs, while those with aura have higher thickness than HCs and those without aura.

* Headache and Thickness of left total hemisphere: Positive MR effect. Headache side thickness is lower than the non-headache side.

* Headache and Thickness of INS dot R: Positive MR effect. Three studies show migraineurs (with or without aura) have lower thickness than H Cs.

* Headache and Thickness of SFG dot R: Positive MR effect. Three studies show migraineurs with visual aura, patients with WMHs, and children with multisite pain have lower thickness than H Cs.

* Headache and Thickness of right total hemisphere: Positive MR effect. Headache side thickness is lower than the non-headache side.

* Headache and Thickness of SMG dot L: Negative MR effect. Pediatric migraineurs and those without aura have lower thickness than HCs, while those with aura have higher thickness.

* Pain and Cortical surface area of STG dot R: Negative MR effect. Chronic migraineurs have lower surface area than H Cs, while another study shows migraineurs have higher surface area.

* Sciatica and Cortical surface area of STG dot R: Negative MR effect. Chronic sciatica patients have higher surface area than H Cs.

Navigate back to Table 1..
